# Investigations into the Effects of pH on Quantitative Measurements of Lactate in Biological Media Using ATR-FTIR Spectroscopy

**DOI:** 10.3390/molecules25163695

**Published:** 2020-08-13

**Authors:** Nystha Baishya, Mohammad Mamouei, Karthik Budidha, Meha Qassem, Pankaj Vadgama, Panayiotis A. Kyriacou

**Affiliations:** 1 Research Center for Biomedical Engineering, University of London, Northampton Square, London EC1V 0HB, UK; mohammad.mamouei@city.ac.uk (M.M.); karthik.budidha@city.ac.uk (K.B.); meha.qassem@city.ac.uk (M.Q.); p.kyriacou@city.ac.uk (P.A.K.); 2 Interdisciplinary Research Centre (IRC) in Biomedical Materials, Queen Mary University of London, Mile End Road, London E1 4NS, UK; p.vadgama@qmul.ac.uk

**Keywords:** lactate, lactic acid, infrared spectroscopy, ATR, sepsis

## Abstract

Quantification of lactate/lactic acid in critical care environments is essential as lactate serves as an important biochemical marker for the adequacy of the haemodynamic circulation in shock and of cell respiration at the onset of sepsis/septic shock. Hence, in this study, ATR-FTIR was explored as a potential tool for lactate measurement, as the current techniques depend on sample preparation and fails to provide rapid response. Moreover, the effects of pH on PBS samples (7.4, 7, 6.5 and 6) and change in solution conditions (PBS to whole blood) on spectral features were also investigated. A total 189 spectra from five sets of lactate containing media were obtained. Results suggests that lactate could be measured with more than 90% accuracy in the wavenumber range of 1500–600 cm${}^{-1}$. The findings of this study further suggest that there exist no effects of change in pH or media, when estimating lactate concentration changes in this range of the Mid-IR spectral region.

## 1. Introduction

Lactate is an averred haemodynamic marker in critical care indicating the adequacy of tissue hypoperfusion [1,2,3,4]. The detection and measurement of lactate, together with acid-base balance has been a primary source of interest for researchers for many decades. A combined abnormality in these parameters is associated with an especially high mortality and morbidity in the critical care environment [5,6,7]. Also recently, lactate has been deemed an important parameter to monitor in critically ill adults with Coronavirus Disease 2019 (COVID-19) [8]. Thus, measurement of lactate has never been more essential. In 2018, the Surviving Sepsis Campaign (SSC) [9] had established that lactate levels ≥4 mM, during admission into critical care, is an indication of tissue hypoxia and must be corrected with utmost urgency. Lactate levels have to be measured intermittently, every 4 h, until the levels reduces to ≤2 mM. In a clinical care setting, arterial blood gas (ABG) analysers are considered the gold standard for lactate measurements. These analysers, which requires sampling of arterial blood, utilises photometric or amperometric techniques where the amount of H_2_O_2_ is correlated to lactate during the catalytic oxidation of lactate by LDH. Hence, these techniques are highly dependent on oxygen and chemical interference in a laboratory and are time consuming.

With the recent global pandemic, which has affected 10,446,353 people world-wide and claimed the lives of 511,037 (as of 1 July 2020) [10], the demand for technological advancements in critical care is now towards precision and expeditious results.

Infrared (IR) absorption spectroscopy, as a lactate measurement technique, could potentially address this demand. The Mid-IR (MIR) region of the Electromagnetic Spectrum is predominantly used for small molecules, and extends from 2.5 μm or 4000 cm${}^{-1}$ to ∼50 μm or 200 cm${}^{-1}$. The absorption bands seen in the MIR region are mostly due to intra-molecular fundamental vibrations, and hence can be referenced to particular organic functional groups, such as, carboxylic acids and their derivatives, e.g., ester, aldehyde and ketone groups. So selective identification is possible using this technique. The feasibility of pH determination in-vitro by studying the carbonate/bicoarbonate systems with the help of infrared spectroscopy has already been established by Baldassarre et al. [11]. When used in combination with Attenuated Total Reflection Fourier Transform Infrared (ATR-FTIR) spectroscopy, these technique could provide rapid, robust and stable sample analysis. ATR-FTIR spectroscopy has already been used to detect biochemical constituents of whole blood [12,13,14], as well as other biological fluid components with high accuracy and sensitivity (which is application specific, more than 90% [15] and >70% [16]), suited to clinical decision making even on un-treated samples. However, the major fundamental limitation of this technique is selectivity towards the analyte of concern in a pool of similar chemical constituents, especially as found in whole blood [17]. This limitation, can however be mitigated by using multivariate analysis techniques, such as, Partial Least Squares Regression (PLSR), which is a bi-linear factor model. Such an algorithm uses a handful of Latent Variables (or PLS components) to describe a wide range of data. Models built with these LVs are then used to predict the dependent values, Y from a large number of independent variables, X [18].

Although ATR-FTIR spectroscopy has been used to study and analyse blood components previously, the simultaneous effects of pH and change in media has not been explored in the case of lactate. Hence, this study focuses on predicting lactate concentration from spectra obtained in-vitro by state-of-the-art FTIR spectrometers, with a Horizontal-ATR accessory. The spectra were obtained from solution samples of varying lactate concentration in buffer at pH values of 7.4, 7.0, 6.5 and 6.0 and then whole blood (pH 7.0–7.3). These spectra were then pre-treated using various chemometric tools and lactate concentrations were predicted using PLSR models, first independently for each data-set, and then simultaneously. This was intended to enable in-depth understanding for the inter-dependence of spectral features for lactate concentrations at different pH values and in different media using IR spectroscopy. In this approach, for the first time, the prediction capabilities of infrared absorption spectroscopy have been tested using solutions of varying lactate concentrations in combination with different pH and media.

## 2. Reagents and Materials

Powdered Na-L-Lactate was procured from (Thermo Fisher Scientific, Waltham, MA, USA). A 600 mM stock solution of lactate was prepared in isotonic Phosphate Buffer Saline (PBS) of pH 7.4. This was used to prepare all the samples as mentioned in the next sub-sections.

### 2.1. Lactate in PBS Samples

The stock pH 7.4 lactate solution was diluted into thirty-seven separate aliquots to give lactate concentrations varying from 0–5 mM, at 0.25 mM intervals and 5–20 mM, at 0.5 mM intervals. These concentrations were chosen, in order to match both physiological lactate levels and those which might be found in critical care [19] and in muscle physiology [20].

Additionally, three separate 600 mM stock solutions of lactate were also prepared in isotonic PBS of pH 7.0, 6.5 and 6.0. The pH of the PBS was altered by drop-wise addition of 0.1 M of HCl. Lactate solution samples were again formulated by diluting these stock solutions, separately to similar concentrations, as mentioned before. The concentrations of lactate and pH of these diluted samples were verified using a LM5 Lactate Analyzer from Analox Instrument Limited, Stourbridge, UK and an Orion Star A211 Advanced pH Benchtop Meter Kit, from Thermo Fisher Scientific, Waltham, MA, USA, respectively. All measurements were taken at a room temperature of 24 ${}^{\circ }$C.

Thereby, a total of 148 distinct solution samples of lactate in PBS of varying concentration and pH were prepared.

### 2.2. Lactate in Blood Samples

An additional set of forty-one samples were prepared by incorporating aliquots of an initial lactate stock solution, which was prepared with sheep whole blood in Alsever’s (Alsever’s Solution is an isotonic solution with NaCl, Citric Acid and D-Glucose, which is typically used for preservation and anti-coagulation of whole blood (almost 10 weeks at 2–8 ${}^{\circ }$C).) (TCS Biosciences Ltd., Buckingham, UK). The reason behind this choice of blood was that blood from sheep has normal and temporal variation of lactate with the closest similarity to human blood. This study was approved by the Senate Research Ethics Committee (SREC) at City, University of London (SREC 17-18 05 6ii 27.06.2018).

Lactate Stock Solution, was diluted to different concentrations of lactate 0 to 10 mM in separate vials of 30 mL each using PBS at room temperature and 7.4 pH. Thereafter, each of 1 mL of the prepared lactate and PBS samples were introduced in the vials containing 19 mL of blood to obtain 41 samples of varying lactate concentrations of 20 mL each. These samples, when measured using the ABL 825 from (Radiometer UK Limited, Crawley, West Sussex, UK), at 25 ${}^{\circ }$C, indicated the range of lactate concentrations to be 4.5 to 13.8 mM with intervals of 0.5 mM with pH levels between 7.1 to 7.3.

## 3. FTIR Spectrometry

Spectra from 2000–500 cm${}^{-1}$ (5000–20,000 nm) were obtained using the Spectrum Two spectrometer (Perkin Elmer, Waltham, MA, USA) for the PBS samples and Frontier FTIR spectrometer (Perkin Elmer, Waltham, MA, USA) for the whole blood samples. The two different spectrometers were used in order to aid in the further understanding of the effects of different instruments on the spectra. Spectra were collected at 0.5–1 cm${}^{-1}$ data intervals and spectral resolution was maintained at 4 cm${}^{-1}$ in the Spectrum Two, as this spectrometer was fitted with an internal fixed J-stop (Jacquinot-stop) of 8.94 mM. However, for the Frontier FTIR, the spectral resolution was maintained at 2 cm${}^{-1}$ and the variable J-stop (Jacquinot-stop) was kept at 3.16 mM at 4000 cm${}^{-1}$ in order to obtain a better throughput.

Furthermore, in order to achieve better Signal-to-Noise Ratio (SNR), multiplexing of 20 scans per sample, with scan speed of 0.2 cm s${}^{-1}$ was adopted for both instruments. Thereby, 70 μL of each sample was exposed directly in the ZnSe crystal of the HATR accessory (Pike Technologies Inc., Madison, WI, USA) for spectra collection. The HATR had a trough Zinc Selenide (ZnSe) crystal of 4 mM thickness, 80 mM length, 2.4 Refractive Index and allowed 10 internal reflections per measurement. The same accessory was used for both the instruments.

Background scans with the empty crystal was taken every 2 samples or every 20 min during each study. Thus, a total of 189 spectra were obtained, which were then pre-treated and analysed.

### Spectral Pre-Processing and Analysis

The five different spectral data-sets of pH values of 7.4, 7.0, 6.5 and 6.0 and whole blood were pre-treated, separately, using various chemometric techniques. This range of pH was chosen, as the normal range of pH in human blood is 7.35 to 7.45 and tends to go towards 7 during severe acidemia and during exercise for an untrained person could drop to 6.94 [21]. Visualization and analysis of all the data-sets were performed in Matlab R2020a, (Mathworks^TM^, Natick, MA, USA). The following techniques were applied in succession for each data-set:

- Spectral Subtraction: The base spectra (0 mM for the PBS data-sets and 4.5 mM for the whole blood data-set) were subtracted from the rest of the spectra in each data-set.
- Extended Multiplicative Scatter Correction (EMSC) with quadratic polynomial baseline correction was used in order to reduce any underlying multiplicative effects on each data-set.
- Savitzky Golay filtering was then used for smoothing and enhancing the absorption peaks in the spectra. The Polynomial Order, Derivative and Window Length of the SG filters used for the PBS data-sets were 2, 1 and 101 and for the whole blood data-set was 2, 1 and 55, respectively. These parameters were identified for each data-set as a trade-off between noise suppression and feature enhancement in the plots through visual inspection.

These pre-treated spectra were analysed using linear regression on observed peaks to confirm the presence of lactic acid and lactate in each data-set. This was then followed by Partial Least Square Regression (PLSR) analysis for each data-set. Model validation was carried out via cross-validation using jackknife (or leave-one-out) approach, in which each spectrum is iteratively held out as a test spectrum and would be predicted using the training model built by all the other spectra (training set). This process would be repeated N times such that every time a different test spectra would be left-out, N being the total number of spectrum in that particular data-set.

The number of LVs, which were used to build the PLS models were determined by the Predicted Residual Sum of Squares (PRESS) for every set. PRESS was used as this was a random model problem and an optimum number of LVs had to be chosen for better predictive ability of the PLS models, which is the smallest value [22]. The predicted concentrations of lactate from these PLS models were then compared to the actual concentrations obtained by the commercial lactate analysers. The coefficient of determination (R${}^{2}$), together with the Root Mean Squared Error of Cross Validation (RMSECV), were then used to understand the accuracy of prediction.

## 4. Results and Discussion

### 4.1. Visual Inspection

The raw spectra from all five data-sets had similar appearance, and no discernible change could be observed due to lactate concentration changes in any of the data-sets. However, after pre-treatment, identical minor peaks could be seen in all the data-sets. These observed peaks could be assigned to fundamental modes of vibration of the functional groups C=O, C-H and O-H. Cassanas et al. [23], in 1991, identified the presence of lactate ion vs lactic acid in the IR region by assigning specific peaks to the fundamental modes of vibration. These were later ascertained by Ube et al. in 2017 [24] in a similar study, where the effects of pH (2.66–1.59) were seen in solution samples at a constant lactate concentration in the Infrared region. The assignment of the bands/peaks were then done using Density Functional Theory (DFT) calculations and were consistent with the previous study. Both these groups came to the conclusion that the changes in the spectra occur because of the ionization of lactic acid to lactate. However, the change of pH or medium from buffer to whole blood, did not have an effect on spectral features of lactate. This indicates that there was no additional distorting effect due to binding interaction with either protein or cations, or if it did occur it was too small to be seen. At the pH values studied, there would have been negligible unionized lactic acid present. The cation common to both sample types is Na${}^{+}$, so it is the prime candidate for any additional lactate ion interaction that could have led to the independent peaks assignable to lactate. Carboxylate ion association with divalent cations is well understood, but assumed not to occur with monovalent cations. Remarkably, there is previous spectroscopic evidence of -COO${}^{-}$… Na${}^{+}$ bonding interaction in aqueous solution which has yet to be recognized in biological samples. This would explain our peaks, and their independence from pH; in our studies only HCl was added to change pH. [25]. Furthermore, studies by Petibois et al. [14,26], in the same spectral region for human plasma samples, were able to identify two more peaks, corresponding to lactate/lactic acid from a pool of other analytes. The observed peaks were: 1399 cm${}^{-1}$ and 1127 cm${}^{-1}$.

**Figure 1:**
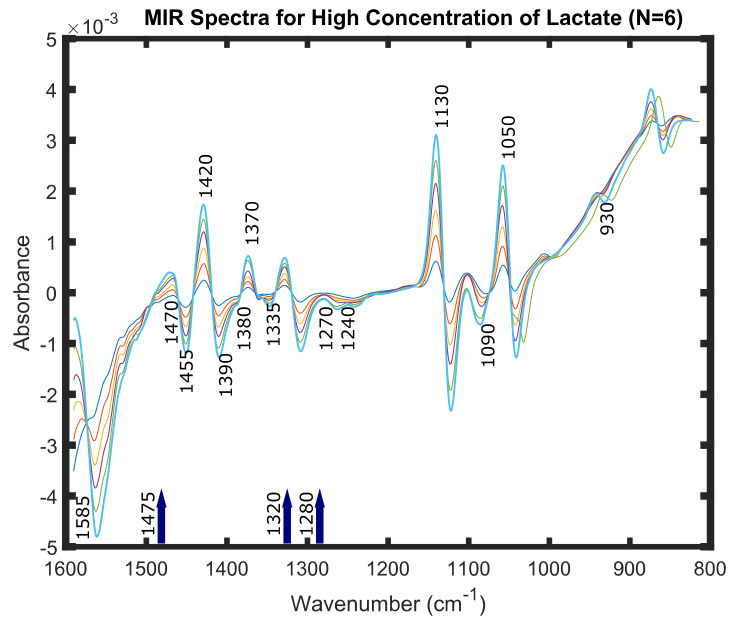
Peaks of interest in the MIR spectral region 800–1600 cm${}^{-1}$, pertinent to high lactate concentration changes. The set of six spectra of very high concentration of lactate in PBS (100 (dark green), 200 (orange), 300 (yellow), 400 (red), 500 (light green) and 600 mM (blue)) were obtained by ATR-FTIR spectroscopy and pre-processing was performed. The pre-processing included: spectral subtraction of 0 mM lactate spectra, EMSC (quadratic polynomial) and Savitzky-Golay filtering (Polynomial Order = 2, Derivative = 2 and Window Length = 101). The peaks 860 cm${}^{-1}$, 930 cm${}^{-1}$, 1050 cm${}^{-1}$, 1090 cm${}^{-1}$, 1130 cm${}^{-1}$, 1240 cm${}^{-1}$, 1270 cm${}^{-1}$, 1280 cm${}^{-1}$, 1320 cm${}^{-1}$, 1335 cm${}^{-1}$, 1370 cm${}^{-1}$, 1380 cm${}^{-1}$, 1390 cm${}^{-1}$, 1420 cm${}^{-1}$, 1470 cm${}^{-1}$, 1475 cm${}^{-1}$ and 1585 cm${}^{-1}$ could be visibly seen in the spectra.

A few of these peaks can be recognised in Figure 1. This figure represents six spectra of very high concentrations of lactate; 600 mM, 500 mM, 400 mM, 300 mM, 200 mM and 100 mM in PBS at pH of 7.4. These spectra were obtained and pre-processed by the same techniques as mentioned in the previous section. Spectra of these concentrations were taken solely for the purpose of visualization of the peaks for lactate ion and lactate containing compounds in the Mid-IR spectral region, as they were not visible distinctly for lower concentrations of lactate.

### 4.2. Linear Regression

Once the peaks were identified in the spectra, linear regression was performed on all the peaks independently, for all five data-sets. The fit of the absorbance values (or peak heights) gave an understanding of the linear correlations between the observed peaks and concentration changes of lactate. Table 1 presents the results of the linear regression on observed peaks, which were reported in the previous section.

The observed peaks, 1725 cm${}^{-1}$ and 1585 cm${}^{-1}$, lie in the sub-region (2000–1500 cm${}^{-1}$), which manifests double bonds within the infrared region of the Electromagnetic Spectrum. These could be assigned to the carbonyl of the carboxylic group (C=O) in the lactate molecule, lactate being an alpha hydroxy acid (AHA) contains one (C=O) group. It can be clearly seen from Table 1, that the change of pH or medium from buffer to whole blood, does not have an effect on measured concentrations of lactate; which remained linearly correlated.

The next spectral range under investigation is the *fingerprint* region (1500–600 cm${}^{-1}$), which has specific signatures of the lactate molecule (both ion and acid form) [23]. The peaks 1475 cm${}^{-1}$, 1455 cm${}^{-1}$, 1380 cm${}^{-1}$, 1130 cm${}^{-1}$, 1050 cm${}^{-1}$ and 930 cm${}^{-1}$ are relative to the lactic acid whereas those at 1470 cm${}^{-1}$, 1370 cm${}^{-1}$, 1125 cm${}^{-1}$, 1045 cm${}^{-1}$ and 860 cm${}^{-1}$ are the same modes but in the lactate ion. These peaks could mostly be assigned to $CH_{3}$ bending vibrations (symmetric and asymmetric) while, 1050 cm${}^{-1}$ arises due to the CH stretching of $C-CH_{3}$ group. The peaks, 1420 cm${}^{-1}$, 1240 cm${}^{-1}$, 1130 cm${}^{-1}$ and 1090 cm${}^{-1}$ could arise in the spectra due to the O-H bending and C-O stretching bonds. All these observed peaks, in this region showed linear correlations across all five data-sets.

The absorbance values for each concentration of lactate could be seen as varying either as an increasing or decreasing trend as depicted by the coefficients of regression (given as positive or negative) in each peak. In order to assess if changes in the concentration of lactate cause statistically significant changes in the absorbance at the identified wavelengths, a hypothesis testing was performed by regressing the concentration of lactate on the absorbance values at different wavelengths using the equation

(1) $$y_{i}=beta_{0}+beta_{1}\times x_{wi}$$

where, $x_{wi}$ is absorbance at wavelength wi, $Y_{i}$ is the concentration of lactate and $beta_{0}$ and $beta_{1}$ are the coefficients. ($beta_{1}$ and standard errors (SE) are presented in parenthesis in Table 1). This was performed in order to test the hypothesis of each individual wavelength. The results suggests that there exist signatures of lactate ion and lactate containing compounds in all five solution samples. The *p*-values ^5^, ^6^, ^7^ and ^8^ depict the statistical significance markers 0.05, 0.005, 0.0005 and 0.00005, respectively.

### 4.3. Building PLS Models

The entire spectral ranges for all the five sets of data were used to construct PLS calibration models, individually. PRESS was used to create the simplest model with least number of LVs. Hence, the number of LVs used for data-sets at pH of 7.4, 7.0, 6.5, 6 and whole blood were 18, 3, 12, 9 and 14, respectively. The models were then evaluated using the leave-one-out cross-validation method independently for all the data-sets. Table 2 shows the Coefficient of Determination (R${}^{2}$) and Root Mean Squared Error of Cross Validation (RMSECV) mM for each data-set, while predicting the Observed (known) lactate concentration versus the Predicted concentrations of lactate in all five data-sets. It could be seen that the Coefficient of Determination (R${}^{2}$) for the data-sets at pH of 7.4, 7.0, 6.5, 6 and whole blood were: 0.96, 0.67, 0.69, 0.85 and 0.92, respectively. The Root Mean Squared Error of Cross Validation (RMSECV) for the same data-sets were: 1.61 mM, 4.21 mM, 5.13 mM, 2.38 mM and 0.82 mM, respectively.

Data-set with pH value of 7.4 and whole blood showed high R${}^{2}$ values and relatively low RMSECV values compared to the other data-sets. However, when PLS regression models were built using only the *‘fingerprint region’*, 1500–600 cm${}^{-1}$, the results were significantly improved. The PRESS, R${}^{2}$ and RMSECV values are as shown in Table 3. The R${}^{2}$ value for the data-sets at pH of 7.4 and whole blood increased by 0.2 and 0.1, respectively while the RMSECV was observed to be almost the same. For data-set at pH of 7.0, the R${}^{2}$ value increased by 0.3 and the RMSECV value decreased from 4.21 mM to 1.34 mM. Similarly, for the data-set at pH of 6.5, the R${}^{2}$ value increased by 0.28 and the RMSECV value decreased from 5.13 mM to 1.48 mM. Again for the data-set at pH of 6, R${}^{2}$ value increased by 0.19, while the RMSECV value was significantly decreased from 6.52 mM to 1.94 mM. The reason behind this could be that the observed peaks seen for lactate ion and lactate containing compounds, in the region 1500–600 cm${}^{-1}$, characterizes these chemical entities more linearly as compared to the other regions in the Mid-IR region.

Hence, it could be inferred that the *‘fingerprint region’* serves as a preferred region for lactate concentration determination. Furthermore, in order to understand the linearity and inter-dependence of pH and change of media for lactate concentration determination, PLSR models were also built using four data-sets. The models were evaluated using the one, which was reserved for the purpose of prediction, by the same models which were built with the rest. The results are as shown in Table 4. The Coefficient of Determination (R${}^{2}$) values for all the iterations were found to be ≥0.90 and the RMSECV value was 1.09 mM. It could be clearly seen that pH and change in media has no effect on the prediction capability of the PLS algorithms and lactate concentrations could be predicted with more than 90% accuracy using any of the data-sets independently as well as combined.

## 5. Conclusions

The influence of pH and change in media, with varying lactate concentrations, in the Mid-IR spectral features were explored in this study. Results from this study clearly indicate that the concentrations of lactate could be linearly correlated to the observed peaks which serve as *fingerprints* for lactate ions and lactate containing compounds in different pH and media. This study also demonstrated the presence of lactate ion in all the solution samples. Regression models using multivariate analysis suggest that in the region 1500–600 cm${}^{-1}$, lactate concentrations could be measured with ≥90% accuracy for each data-set independently. While combining the data-sets for lactate concentration prediction, evidence from this study again established an accuracy of ≥90%. Hence, this study demonstrated for the first time that ATR-FTIR spectroscopy could be used to predict lactate concentrations in solution samples and there exists no effects of pH or media in the spectral features. In conclusion, ATR-FTIR spectroscopy could be utilised as a potential lactate measuring technology in critical care settings. This technique is non-destructive with limited sample preparation because of high throughput capabilities, and offers almost real time measurements of samples as compared to traditional methods. However, this technique still has the limitation of being temperature dependant and needs further processing for interpretation. There is clear potential in extending this technique towards more complex human blood samples analysis in commercial laboratory settings.

## Figures and Tables

**Table 1:** Linear Regression (1) on observed peaks (cm${}^{-1}$) pertinent to lactate ion or lactate containing compounds in solution. (Coefficient and Standard errors in parenthesis. *p*-values depict statistical significance markers 0.05, 0.005, 0.0005 and 0.00005, respectively.)

Observed Peak (cm${}^{-1}$)	Data-Set pH 7.4	Data-Set pH 7.0	Data-Set pH 6.5	Data-Set pH 6.0	Whole Blood Data-Set
1725	−3.0 × 10${}^{5}$(48966)^8^	−2.4 × 10${}^{5}$(65625)^7^	27,634(2.4 × 10${}^{5}$)	57,174(2.7 × 10${}^{5}$)	5(0.46771)^8^
1585	30,243(54,896)^8^	−8581(1.8 × 10${}^{5}$)^8^	−91,434(29,751)^6^	−3.5 × 10${}^{5}$(89821)^7^	5(0.46)^8^
1475	−7.4 × 10${}^{5}$(1.1 × 10${}^{5}$)^8^	−990.5(41469)^8^	3.0 × 10${}^{5}$(59,340)^8^	−9.4 × 10${}^{5}$(2.3 × 10${}^{6}$)^5^	5(0.46)^8^
1470	−7.9 × 10${}^{5}$(1.4 × 10${}^{5}$)^8^	−3969.1(59,054)^6^	1.5 × 10${}^{5}$(51,313)^6^	−1 × 10${}^{6}$(2.5 × 10${}^{5}$)^7^	410.3(4545.6)
1455	1.6 × 10${}^{6}$(6.3 × 10${}^{5}$)^5^	2.8 × 10${}^{5}$(77,146)^7^	−52,431(1.9 × 10${}^{5}$)	−8.4 × 10${}^{5}$(1.4 × 10${}^{5}$)^8^	5(0.46)^8^
1420	−3.9 × 10${}^{5}$(68,005)^8^	−1.6 × 10${}^{5}$(18663)^8^	−69,730(1.7 × 10${}^{5}$)	−7.5 × 10${}^{5}$(3.6 × 10${}^{5}$)^5^	71,863(14,962)^8^
1390	7.8 × 10${}^{5}$(1 × 10${}^{5}$)^8^	−40866(12143)^6^	−5.9351 × 10${}^{5}$(1.2 × 10${}^{5}$)^8^	1.1 × 10${}^{6}$(9.7 × 10${}^{5}$)	22,157(10465)^5^
1380	3.8 × 10${}^{5}$(98408)^7^	1.9 × 10${}^{5}$(85,979)^5^	3.9 × 10${}^{5}$(1.3 × 10${}^{5}$)^6^	1.2 × 10${}^{6}$(3.4 × 10${}^{5}$)^7^	16,098(9087.9)
1399	1.2 × 10${}^{6}$(2.4 × 10${}^{5}$)^8^	−47,402(11717)^7^	−1.4 × 10${}^{5}$(22754)^8^	−4.5 × 10${}^{6}$(8.6 × 10${}^{5}$)^8^	5(0.46)^8^
1370	2.7 × 10${}^{5}$(76461)^6^	−3.8 × 10${}^{5}$(1.4 × 10${}^{5}$)^6^	−1.8 × 10${}^{5}$(30,127)^8^	7.1 × 10${}^{5}$(2.8 × 10${}^{5}$)^5^	10,893(8253.3)
1335	−5.7 × 10${}^{5}$(8.51 × 10${}^{5}$)	4.2 × 10${}^{5}$(87,684)^8^	−6.6 × 10${}^{5}$(2.4 × 10${}^{5}$)^5^	1 × 10${}^{6}$(4.4 × 10${}^{5}$)^5^	5(0.46)^8^
1320	−2.7 × 10${}^{6}$(9.5 × 10${}^{5}$)^6^	−6.1 × 10${}^{5}$(2.1 × 10${}^{5}$)^6^	−2.7 × 10${}^{5}$(2.2 × 10${}^{5}$)	2.2 × 10${}^{6}$(1.8 × 10${}^{5}$)^8^	−52,620(22,503)^5^
1280	−4.3 × 10${}^{5}$(1.3 × 10${}^{5}$)^6^	−8.9 × 10${}^{5}$(4.1 × 10${}^{5}$)^5^	1.8 × 10${}^{5}$(41,833)^8^	1.4 × 10${}^{6}$(1.6 × 10${}^{5}$)^8^	−11,351(3960.9)^6^
1270	−3.7 × 10${}^{5}$(83,646)^8^	−4 × 10${}^{5}$(1.4 × 10${}^{5}$)^5^	1.4 × 10${}^{5}$(42,949)^5^	1.4 × 10${}^{6}$(1.8 × 10${}^{5}$)^8^	−8693.9(3599.5)^5^
1240	1.2 × 10${}^{6}$(4 × 10${}^{5}$)^6^	2.5 × 10${}^{5}$(94227)^5^	7.5 × 10${}^{5}$(1.5 × 10${}^{5}$)^8^	2.5 × 10${}^{6}$(1.9 × 10${}^{5}$)^8^	−8683.7(4387.9)^5^
1130	−3.5 × 10${}^{5}$(41622)^8^	−1.2 × 10${}^{6}$(2.1 × 10${}^{5}$)^8^	−5.6 × 10${}^{5}$(3.2 × 10${}^{5}$)^8^	−4.6 × 10${}^{5}$(1.3 × 10${}^{5}$)^7^	−7586.8(3326.5)^5^
1127	−3.2 × 10${}^{5}$(45268)^8^	−8.3 × 10${}^{5}$(1.1 × 10${}^{5}$)^8^	−1.1 × 10${}^{5}$(1.2 × 10${}^{5}$)^8^	−3.9 × 10${}^{5}$(1.2 × 10${}^{5}$)^6^	5(0.46)^8^
1125	−3 × 10${}^{5}$(47,297)^8^	−7.9 × 10${}^{5}$(79,641)^8^	77,722(10,984)^8^	−3.4 × 10${}^{5}$(1.2 × 10${}^{5}$)^5^	5(0.46)^8^
1090	−1 × 10${}^{5}$(25642)^7^	1 × 10${}^{5}$(1.4 × 10${}^{5}$)^5^	1 × 10${}^{6}$(2.45 × 10${}^{5}$)^7^	11,957(1.1 × 10${}^{5}$)	−12,703(3735)^6^
1050	2 × 10${}^{5}$(39282)^8^	2.2 × 10${}^{5}$(61,645)^7^	4.8 × 10${}^{5}$(92,032)^8^	1.9 × 10${}^{5}$(1.3 × 10${}^{5}$)	−3232.2(4430.4)
1045	1.3 × 10${}^{5}$(29219)^8^	42,252(33,079)^5^	−92,317(14,288)^8^	1.6 × 10${}^{5}$(1 × 10${}^{5}$)	5(0.46)^8^
930	−2 × 10${}^{5}$(48,867)^7^	2.9 × 10${}^{5}$(2.6 × 10${}^{5}$)	3.3 × 10${}^{5}$(3.4 × 10${}^{5}$)	3.8 × 10${}^{5}$(1.9 × 10${}^{5}$)^5^	−3404.3(1586.9)^5^
860	−2.9 × 10${}^{5}$(61216)^8^	1.5 × 10${}^{5}$(1.4 × 10${}^{5}$)^5^	−1.1 × 10${}^{5}$(26,365)^8^	1.5 × 10${}^{5}$(1.7 × 10${}^{5}$)	−3342.4(1295.6)^5^

**Table 2:** The Coefficient of Determination (R${}^{2}$) and Root Mean Squared Error of Cross Validation (RMSECV) (mM) for each data-set, independently.

Observed Peak (cm${}^{-1}$)	Data-Set pH 7.4	Data-Set pH 7.0	Data-Set pH 6.5	Data-Set pH 6.0	Whole Blood Data-Set
Coefficient of Determination
(R${}^{2}$)	0.96	0.67	0.69	0.85	0.92
Root Mean Squared Error
of Cross Validation
(RMSECV) (mM)	1.16	4.21	5.13	2.38	0.82

**Table 3:** The Coefficient of Determination (R${}^{2}$) and Root Mean Squared Error of Cross Validation (RMSECV) (mM) for each data-set, independently, in the region 1500–600 cm${}^{-1}$.

Observed Peak (cm${}^{-1}$)	Data-Set pH 7.4	Data-Set pH 7.0	Data-Set pH 6.5	Data-Set pH 6.0	Whole Blood Data-Set
Latent Variables (LV) from
Predicted Residual Sum of Squares (PRESS)	12	13	2	20	11
Coefficient of Determination
(R${}^{2}$)	0.98	0.97	0.97	0.99	0.93
Root Mean Squared Error
of Cross Validation
(RMSECV) (mM)	1.16	1.34	1.48	1.94	0.78

**Table 4:** Linear Regression results of the pH and whole blood data-sets used to build predictive models.

Data-Sets Used	Data-Set Predicted	No. of LVs Used	R${}^{2}$	RMSECV (mM)
pH 7.0, pH 6.5, pH 6 and whole blood	pH 7.4	18	0.90	1.09
pH 7.4, pH 6.5, pH 6 and whole blood	pH 7.0	18	0.93	1.09
pH 7.4, pH 7, pH 6 and whole blood	pH 6.5	18	0.95	1.09
pH 7.4, pH 7, pH 6.5 and whole blood	pH 6	18	0.92	1.09
pH 7.4, pH 7, pH 6.5 and pH 6	whole blood	18	0.97	1.09

## References

[1] Burstein B., Vallabhajosyula S., Ternus B., Barsness G.W., Kashani K., Jentzer J.C. (2020). The Prognostic Value of Lactate in Cardiac Intensive Care Unit Patients with Cardiac Arrest and Shock. Shock.

[2] Gutiérrez H.B., Concepción Y.A., Pérez J.S., Lara Y.D., López F.M.R., Contreras P.R. (2020). Prognostic Value of Serum Lactate Levels in Critically Ill Patients in an Intensive Care Unit. J. Crit. Care Med..

[3] Han K.S., Kim S.J., Lee E.J., Park K.Y., Lee J.Y., Lee S.W. (2019). Impact of rapid lactate clearance as an indicator of hemodynamic optimization on outcome in out-of-hospital cardiac arrest: A retrospective analysis. PLoS ONE.

[4] Fuller B.M., Dellinger R.P. (2012). Lactate as a hemodynamic marker in the critically ill. Curr. Opin. Crit. Care.

[5] He Y., Ong J., Ong S. (2019). Refractory Lactic Acidosis and an Approach to its Management—A Case Report. J. Crit. Care Med..

[6] Allen M. (2011). Lactate and acid base as a hemodynamic monitor and markers of cellular perfusion. Pediatr. Crit. Care Med..

[7] Suetrong B., Walley K.R. (2016). Lactic Acidosis in Sepsis: It’s Not All Anaerobic: Implications for Diagnosis and Management. Chest.

[8] Alhazzani W., Møller M.H., Arabi Y.M., Loeb M., Gong M.N., Fan E., Oczkowski S., Levy M.M., Derde L., Dzierba A. (2020). Surviving Sepsis Campaign: Guidelines on the management of critically ill adults with Coronavirus Disease 2019 (COVID-19). Intensive Care Med..

[9] Levy M.M., Evans L.E., Rhodes A. (2018). The Surviving Sepsis Campaign Bundle: 2018 Update. Crit. Care Med..

[10] https://www.ecdc.europa.eu/en/geographical-distribution-2019-ncov-cases.

[11] Baldassarre M., Barth A. (2014). The carbonate/bicarbonate system as a pH indicator for infrared spectroscopy. Analyst.

[12] Paraskevaidi M., Hook P.D., Morais C.L.M., Anderson J.R., White R., Martin-Hirsch P.L., Peffers M.J., Martin F.L. (2020). Attenuated total reflection Fourier-transform infrared (ATR-FTIR) spectroscopy to diagnose osteoarthritis in equine serum. Equine Vet. J..

[13] Santos M.C.D., Nascimento Y.M., Araújo J.M.G., Lima K.M.G. (2017). ATR-FTIR spectroscopy coupled with multivariate analysis techniques for the identification of DENV-3 in different concentrations in blood and serum: A new approach. R. Soc. Chem. Adv..

[14] Petibois C., Cazorla G., Cassaigne A., Déléris G. (2001). Plasma protein contents determined by Fourier-transform infrared spectrometry. Clin. Chem..

[15] Ferreira I.C.C., Aguiar E.M.G., Silva A.T.F., Santos L.L.D., Cardoso-Sousa L., Araújo T.G., Santos D.W., Goulart L.R., Sabino-Silva R., Maia Y.C.P. (2020). Attenuated Total Reflection-Fourier Transform Infrared (ATR-FTIR) Spectroscopy Analysis of Saliva for Breast Cancer Diagnosis. J. Oncol..

[16] Maitra I., Morais C.L.M., Lima K.M.G., Ashton K.M., Date R.S., Martin F.L. (2019). Attenuated total reflection Fourier-transform infrared spectral discrimination in human bodily fluids of oesophageal transformation to adenocarcinoma. Analyst.

[17] Perez-Guaita D., Garrigues S. (2014). Infrared-based quantification of clinical parameters. TrAC Trends Anal. Chem..

[18] Kourti T. (2009). Multivariate Statistical Process Control and Process Control, Using Latent Variables.

[19] Webb A.L., Kramer N., Rosario J., Dub L., Lebowitz D., Amico K., Leon L., Stead T.G., Vera A., Ganti L. (2020). Delta Lactate (Three-hour Lactate Minus Initial Lactate) Prediction of In-hospital Death in Sepsis Patients. Cureus.

[20] Goodwin M.L., Harris J.E., Hernández A., Gladden L.B. (2007). Blood Lactate Measurements and Analysis during Exercise: A Guide for Clinicians. J. Diabetes Sci. Technol..

[21] Ghoneim M.T., Nguyen A., Dereje N., Huang J., Moore G.C., Murzynowski P.J., Dagdeviren C. (2019). Recent Progress in Electrochemical pH-Sensing Materials and Configurations for Biomedical Applications. Chem. Rev..

[22] Abdi H., Williams L.J. (2013). Partial Least Squares Methods: Partial Least Squares Correlation and Partial Least Square Regression. Methods in Molecular Biology.

[23] Cassanas G., Morssli M., Fabrègue E., Bardet L. (1991). Vibrational spectra of lactic acid and lactates. J. Raman Spectrosc..

[24] Ube T., Yoneyama Y., Ishiguro T. (2017). In situ Measurement of the pH-dependent Transmission Infrared Spectra of Aqueous Lactic Acid Solutions. Anal. Sci..

[25] Sthoer A., Hladílková J., Lund M., Tyrode E. (2019). Molecular insight into carboxylic acid–alkali metal cations interactions: Reversed affinities and ion-pair formation revealed by non-linear optics and simulations. Phys. Chem. Chem. Phys..

[26] Petibois C., Melin A.M., Perromat A., Cazorla G., Déléris G. (2000). Glucose and lactate concentration determination on single microsamples by Fourier-transform infrared spectroscopy. J. Lab. Clin. Med..

